# Comparative pharmacognosy and secondary metabolite analysis of *Balanophorae* herbs from different sources

**DOI:** 10.1186/s41065-024-00323-1

**Published:** 2024-06-21

**Authors:** Xueyan Zhao, Lihui Zheng, Qingxin Shi, Yuqi Lin, Zhaoxiang Zeng, Chengwu Song, Shuna Jin, Ling Xiao

**Affiliations:** 1 School of Pharmacy, Hubei University of Chinese Medicine, Wuhan, Hubei 430065 China; 2 Hubei Institute for Drug Control, Hubei Engineering Research Center for Drug Quality Control, NMPA Key Laboratory of Quality Control of Chinese Medicine, Wuhan, Hubei 430075 China; 3 School of Basic Medical Sciences, Hubei University of Chinese Medicine, HuangJiaHu West Road 16, Wuhan, Hubei 430065 China; 4 Hubei Shizhen Laboratory, Wuhan, Hubei 430065 China

**Keywords:** *Balanophorae*, Pharmacognosy, Secondary metabolites, LC-MS, Targeted metabolomics

## Abstract

The *Balanophorae* are not only traditional Chinese herbal medicines but also functional foods with diverse sources. This study aimed to distinguish pharmacognostic characteristics and secondary metabolites among different species of *Balanophorae*. Eight species of *Balanophorae* herbs were harvested, including 21 batches with 209 samples. Ultra-high-performance liquid chromatography-quadrupole time-of-flight mass spectrometry was used to analyze secondary metabolites of *Balanophorae* from 21 sources. Targeted metabolomic analysis was performed to compare differences among the groups. *Rhopalocnemis phalloide* and *B. indica* can be identified by their pharmacognostic characteristics. Then, 41 secondary metabolites were identified or characterized in the mixed extracts of the 209 samples, mainly phenolic acids, flavonoids, and their derivatives. The distribution of these secondary metabolites revealed apparent differences among different species. In addition, targeted metabolomic analysis suggested that the secondary metabolite profiles of seven species of *Balanophorae* showed noticeable differences, and differences were also observed among different growing regions. Finally, five important metabolic markers were screened to successfully distinguish *B. laxiflora*, *B. harlandii*, and *B. polyandra*, including three phenolic acids and two flavonoids. This is the first study to systematically compare both the morphology and secondary metabolites among different sources of *Balanophorae*, which could provide effective information for identifying diverse species.

**Supplementary Information:**

The online version contains supplementary material available at 10.1186/s41065-024-00323-1.

## Introduction

*Balanophorae* are typically parasitic plants with no green leaves and their aerial parts resemble mushrooms [1]. Among these, the only two genera that grow in China are *Balanophora* and *Rhopalocnemis*, many of which have been used in folk medicine for thousands of years without a uniform quality standard. They are also consumed as functional foods with various bioactive substances in southeast China and are stewed with beef and mutton [2]. In traditional Chinese medicine, most species of *Balanophorae* have a wide range of functions, mainly focusing on acesodyne, hemostatic efficacy, liver protection, and nourishing kidney [3–5]. However, different species have diverse medicinal uses. For example, *Rhopalocnemis phalloides* (RP) is often used for treating the common cold and injuries from falls and as a tonic remedy [6]. *Balanophora polyandra* (BP) is used as an antipyretic, antidote, hemostatic, and blood tonic [7]. *Balanophora laxiflora* (BL) is traditionally used to treat cough, metrorrhagia, and hemorrhoids [8], and *Balanophora harlandii* (BH) is used to treat syphilis and herpes [9].

The morphological characteristics of *Balanophorae* plants have been recorded in the Flora of China and can be used to identify different species [5]. However, with changes in the ecological environment and variation in species, we found that some species of *Balanophora* in our collection were easily confused owing to their pharmacognostic similarities. Thus, a rapid and accurate method for accurately identifying *Balanophora* needs to be developed. Several small organic molecules known as secondary metabolites exhibit excellent biological activities [10]. Flavonoid, phenols, and triterpenes have been reported to be the main secondary metabolites of *Balanophorae* herbs [8, 11–13]. Plant species and environmental factors influence the biosynthesis and accumulation of secondary metabolites and eventually affect the internal quality of herbs [14, 15]. Secondary metabolite analysis has become an effective approach for identifying plant species [16]. However, the comparative pharmacognostic characteristics of whole dried *Balanophorae* plants and the differences of secondary metabolites among different sources of *Balanophorae* need to be further investigated.

Metabolomics is concerned with the measurement of metabolites and identification of factors that may alter metabolites due to genetic, environmental, or dietary influences [17]. In the context of medicinal plants, metabolomics is widely used to study the production regions, identify plant species, and evaluate and ensure quality control [18–20]. Liquid chromatography coupled with mass spectrometry (LC-MS) is widely employed in plant metabolomic research with high sensitivity, resolution, and specificity [21]. Comprehensive analysis not only revealed the differences among several sources of *Balanophorae* but also helped identify the metabolites of the herb based on LC-MS.

In this study, a comparative pharmacognosy of dried plants from 209 samples of 8 species of *Balanophorae* was performed to reveal their morphological characteristics. Secondary metabolites in 209 samples of *Balanophorae* were identified and characterized using ultra-high-performance liquid chromatography quadrupole time-of-flight mass spectrometry (UPLC-QTOF-MS/MS). The distribution of secondary metabolites revealed differences among the eight species, and a target metabolomics approach was then established to evaluate the metabolite differences among BL, BH, and BP. The distribution of secondary metabolites among the eight species was investigated and compared.

## Experimental

### Materials and regents

To collect samples from as many sources as possible, the collection area included the provinces of Hubei, Guizhou, Sichuan, Yunnan, and Guangxi. The 21 batches fresh *Balanophorae* (BL, BH, BP, *Balanophora subcupularis* (BSu), *Balanophora simaoensis* (BSi), *Balanophora cryptocaudex* (BC), *Balanophora indica* (BI), and RP.

**Fig. 1:**
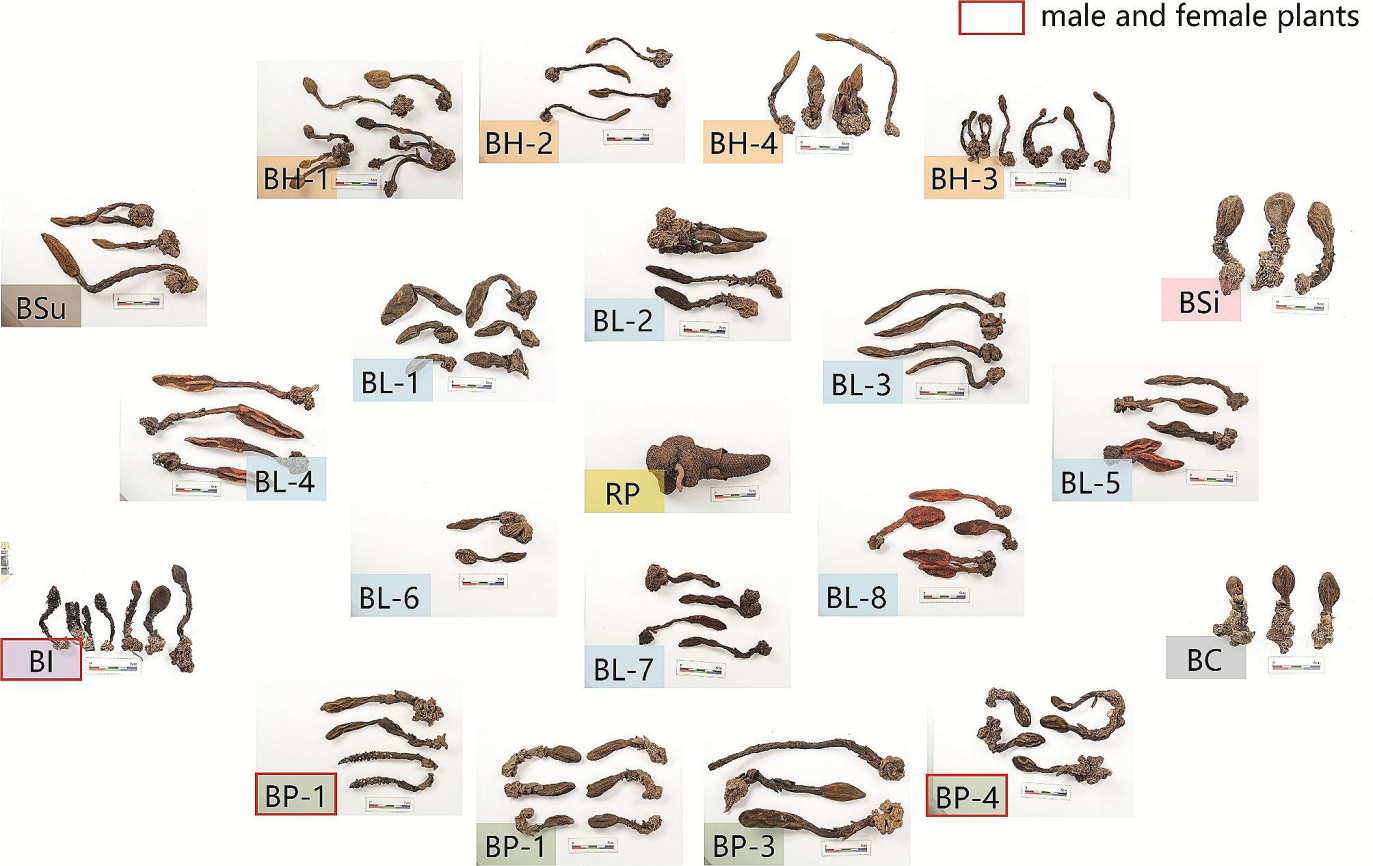
Pictures of dried *Balanophoraceae* plants from 21 sources

Detailed information (plant number, province, region, and regional coordinates) on the 209 collected *Balanophorae* samples is shown in Table 1. All samples were authenticated by Professor Du Wei from Wuhan University, and voucher samples were deposited in the herbarium of Hubei University of Chinese Medicine (No.202,208,116). The whole dried plants of the 21 batches *Balanophorae* herbs are shown in Fig. 1.

**Table 1:** The detailed information of the collected *Balanophorae* samples

Plant Number	Province	Region	Longitude E°	Latitude *N*°
BL-1^a^ (*n* = 10)	Hubei	Enshi City	109.4	30.12
BL-2 ^a^ (*n* = 10)		Lichuan City	108.74	30.19
BL-3 ^a^ (*n* = 10)	Guizhou	Bijie City	105.38	26.77
BL-4 ^a^ (*n* = 10)	Yunnan	Wenshan City	104.24	23.39
BL-5 ^a^ (*n* = 10)		Purchased from Sichuan Lotus Pond Chinese Herbal Medicine Market	-	-
BL-6 ^a^ (*n* = 10)		Purchased from Haozhou Herbal Medicine Market	-	-
BL-7 ^a^ (*n* = 10)	Guangxi	Purchased from Sichuan Lotus Pond Chinese Herbal Medicine Market	-	-
BL-8 ^a^ (*n* = 10)		Purchased from Sichuan Lotus Pond Chinese Herbal Medicine Market	-	-
BSu ^a^ (*n* = 10)	Yunnan	Nujiang Prefecture	98.83	27.39
BH-1 ^a^ (*n* = 10)	Hubei	Shennongjia	110.7	31.75
BH-2 ^a^ (*n* = 10)		Lichuan City	108.74	30.19
BH-3 ^a^ (*n* = 9)	Sichuan	Luzhou City	105.36	28
BH-4 ^a^ (*n* = 10)	Yunnan	Wenshan City	104.2	23.15
BP-1^b^ (*n* = 10)	Hubei	Shennongjia	110.43	31.67
BP-2 ^a^ (*n* = 10)		Xianfeng Country	109.08	29.95
BP-3 ^a^ (*n* = 10)		Shennongjia	110.35	31.43
BP-4^b^ (*n* = 10)	Yunnan	Wenshan City	104.22	23.19
BSi ^a^ (*n* = 10)	Yunnan	Lincang City	99.26	24.03
BC ^a^ (*n* = 10)	Yunnan	Puer City	100.96	24.3
BI^b^ (*n* = 10)	Yunnan	Lincang City	99.26	24.03
RP ^a^ (*n* = 10)	Yunnan	Wenshan City	103.95	23.48

Abbreviations: BL, *Balanophora laxiflora*; BSu, *Balanophora subcupularis*; BH, *Balanophora harlandii*; BP, *Balanophora polyandra*; BSi, *Balanophora simaoensis*; BC, *Balanophora cryptocaudex*; BI, *Balanophora indica*; RP, *Rhopalocnemis phalloides*

Notes: ^a^ female samples; ^b^ 5 male samples and 5 female samples

HPLC grade methanol was purchased from Fisher Scientific (Waltham, MA). Formic acid (≥ 98%) of analytical grade was obtained from Sinopharm Chemical Reagent Co., Ltd (Shanghai, China). Deionized water was obtained using a Milli-Q water system (Millipore, Bedford, MA, USA). The standards of eriodictyol (≥ 95%), eriodictyol-glucoside-1 (≥ 95%), naringenin, naringenin -glucoside-1, trilobatin, phloridzin, β-Amyrin, β-Amyrin acetate, Lupeol acetate-1, and ellagic acid were purchased from Chengdu Biochem Pure Biotechnology Co., Ltd. An internal standard (IS) of curcumin (98% purity, as verified using HPLC-UV) was prepared.

### Sample preparation and extraction

The dried crude plants from the 209 samples were crushed. A 100 mg powder of individual sample was accurately weighed and transferred into a tube, followed by the addition of 5 mL of 80% aqueous methanol containing IS (final concentration of curcumin was 200ng·mL^− 1^). After vortexing for 1 min, the samples were extracted in an ultrasonic bath for 5 min and centrifuged at 3500 rpm (1863 ×g). The supernatants were carefully removed from the extracts. The extraction process was repeated twice with 5 mL each of 50% aqueous methanol solution and 100% methanol solvent, and the three separate extracts were merged. Finally, the combined extracts were centrifuged at 12,000 rpm (12,830 ×g) for 10 min, and the supernatant was used for LC-MS/MS.

### UPLC-QTOF-MS/MS conditions

The obtained extracts were analyzed using a Waters ACQUITY UPLC M-class system (Waters, Mass, USA) coupled with a Xevo G2-XS QTof system equipped with an electrospray ionization source (Waters).

ACQUITY UPLC BEH C18 column (100 × 2.1 mm, 1.7 μm; Waters, Milford, MA, USA) was used for chromatographic separation. The mobile phase comprised solvents A (0.1% formic acid in water, v/v) and B (methanol). Gradient program was as follows: 0–15 min, 10–60% B; 15–22 min, 60–95% B; 22–28 min, 95–99% B; 28–33 min, 99–99% B; 33–34 min, 99–10% B; 34–38 min, 10–10%B. The main parameters were set as follows: flow rate, 0.30 mL·min^− 1^; oven temperature, 35 ℃; injection volume, 2 µL.

MS was conducted using positive ion electrospray in sensitivity analysis mode. The MS parameters were as follows: source temperature, 100 ℃; desolvation temperature, 500 ℃; cone gas flow, 50 L·h^− 1^; desolvation gas flow, 600 L·h^− 1^; cone voltage, 40 V; capillary voltage, 3 kV. The mass ranges were set at *m/z* 50–1200 Da for a full scan with a scan duration of 1 s. Data were collected in the MS^E^ mode. Leucine enkephalin was used as a calibrator [500 pg·mL^− 1^, (M + H)^+^ at *m/z* 556.2771] to ensure mass accuracy and reproducibility at a flow rate of 20 µL·min^− 1^.

### Data processing, statistical analysis, and metabolite identification

Raw data files acquired from the LC-MS were collected and analyzed using Masslynx V4.1 (Waters, Mass, USA). The resulting output data of retention time, mass-to-charge ratio (*m/z*), and peak area acquired for each sample were subjected to further statistical analysis. Relative quantification was performed using the ratio of the peak area of the compound to that of the IS.

The decadic logarithm (log10 transformation) of the data was used for principal component analysis (PCA) and visualization of the heat map of the 41 identified secondary metabolites. Subsequently, the partial least-squares discriminant analysis (PLS-DA) model and rank sum test were employed to select differential secondary metabolites using the following criteria: variable importance in projection (VIP) > 1 and *P* < 0.05.

Statistical analyses of PCA and PLS-DA were performed using the SIMCA-P 14.1 software (Umetrics AB, Umeå, Sweden). The rank-sum test was conducted using the IBM SPSS Statistics 26 (International Business Machines Corporation, Chicago. Graphs were generated using GraphPad Prism 7.0.

## Results

### Pharmacognosy study

The entire *Balanophorae* plant is composed of both underground and aerial parts; the former is the rhizome, and the latter consists of squama bracts, scape, and spadix. The dried plants of *Balanophorae* from different species have different characteristics (see Table 2).

**Table 2:** Pharmacognostic characteristics of eight species of *Balanophoraceae*

Species	Root	Scape	Female flower
*Balanophora laxiflora*	Brownness or reddish black	Red or brown Few squama bracts	Red or brown Tapering at the top
*Balanophora subcupularis*	Tawny	Brownness Covered with brown alternate squama bracts	Claybank Oblong
*Balanophora harlandii*	Obvious cerebral folds	Sepia Few squama bracts	Yellow or brown Oval or oblong
*Balanophora polyandra*	Densely covered with granular verrucous tumors Star-shaped lenticels	Brownness Some squama bracts	Elongated ovoid Tapering at the base
*Balanophora simaoensis*	Few granular verrucous tumors Star-shaped lenticels	<5 cm in length Covered with squama bracts	Brown-yellow oval
*Balanophora cryptocaudex*	Densely covered with granular verrucous tumors Star-shaped lenticels	<3 cm in length Covered with squama bracts	Brown-yellow oval
*Balanophora indica*	Few star-shaped lenticels Yellow and brown	Sepia Brown squama bracts	Sepia or brown oval
*Rhopalocnemis phalloides*	Thick and smooth	Stubby and barely visible	Covered with thick, angular, peltate squama bracts

The rhizome of BL was brown or reddish-black, and the scape was red or brown with few squama bracts. The female flowers are ovoid, enlarged at the base, and acuminate at the top, which are the most distinctive characteristics of BL compared to other species [22]. The rhizomes of BH showed obvious cerebral fold changes. The scape was sepia with a few squama bracts, and the female flowers were oval or oblong. The scape of BSu is covered with alternating brown squamous bracts. However, the characteristics of female flowers and rhizomes were not significantly different and were similar to those of BL and BH flowers. The BP rhizome is densely covered with granular verrucous tumors and has star-shaped lenticels. Female flowers were oblong and acuminate at the bottom, in contrast to BL, which is the most distinctive characteristic of BP. The rhizomes of BSi were covered with a few granular verrucous tumors and star-shaped lenticels. The scape was short, thick, 3–5 cm long, and covered with squama bracts. The female flower is elongated and ovoid. BC and BSi were similar in appearance and difficult to distinguish. The rhizomes of BI were yellow-brown, with a few star-shaped lenticels. The scape was sepia with brown squamous bracts, and the female flowers were oval.

The appearance of RP was quite different from that of *Balanophora* plants. The rhizomes are thick and relatively smooth. The scape is stubby and barely visible. Female flowers were covered with thick, angular, and peltate squamous bracts.

Although the eight species showed different characteristics, some species, such as BSi and BC, were still difficult to distinguish owing to their similarity in appearance. In addition, they are often purchased as decoction pieces or prepared medicines, making it more difficult to trace the original appearance of Balanophorae plants. Therefore, exploring the qualitative and quantitative differences in secondary metabolites among the 21 batches of *Balanophorae* obtained from different sources was necessary.

### Analysis of secondary metabolites based on UPLC-QTOF-MS/MS

**Fig. 2:**
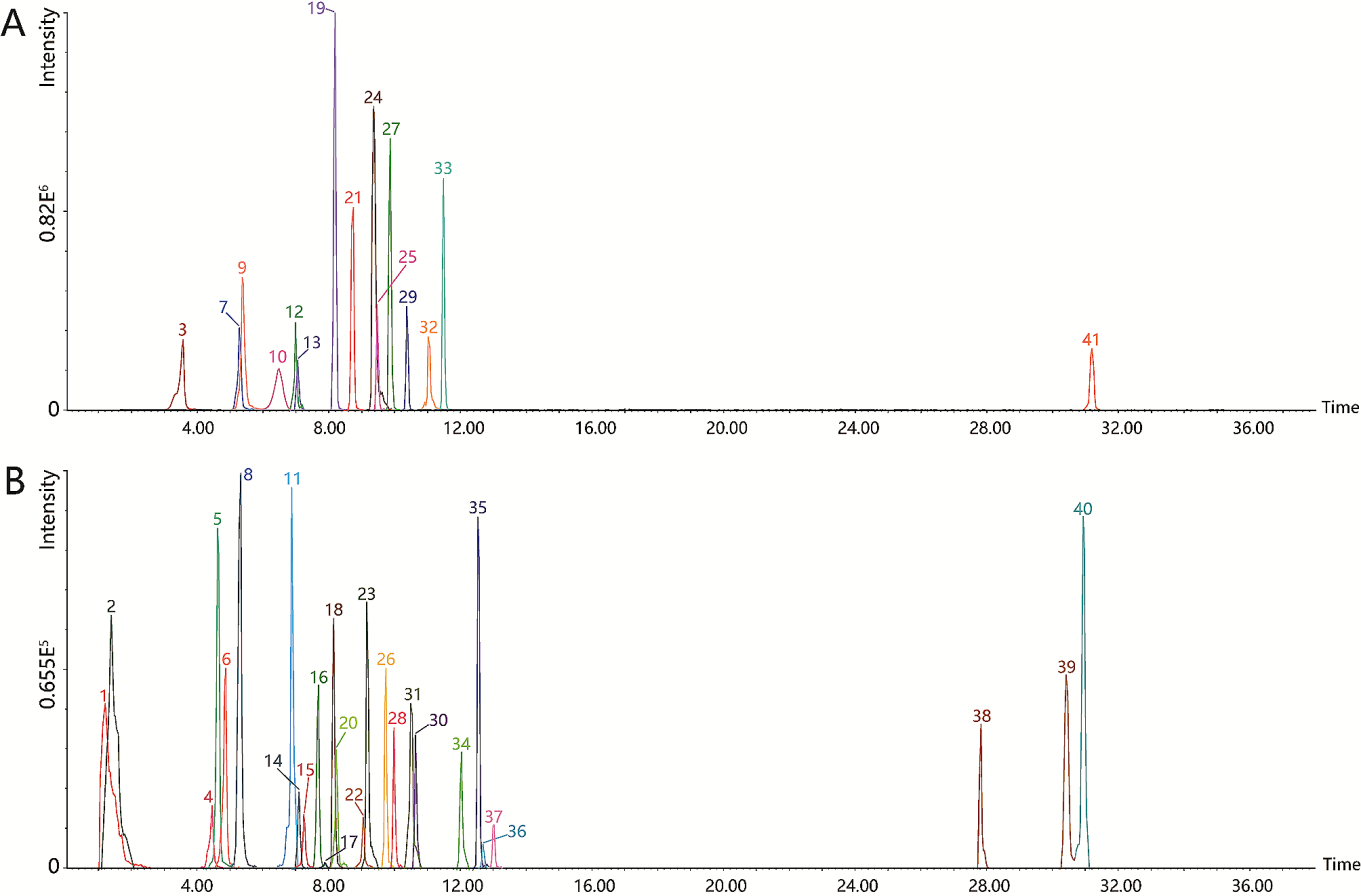
Integration of individual peak in extracted ion chromatograms (EICs) of the 41 compounds using ultra-high-performance liquid chromatography-quadrupole time-of-flight mass spectrometry. **A** and **B** are EICs of compounds with different intensity, and they are shown separately so that the EICs of each compound could be listed. Peak numbers of compounds are consistent with those in Table 2

As shown in Table S1, 41 compounds were identified or characterized in the mixed extracts of 209 samples based on standards and relevant literature [3, 23–27]. The 41 compounds included 17 phenolic acids and their derivatives, 19 flavonoids and their derivatives, and 5 others. The extracted ion chromatograms (EIC) of each compound are shown in Fig. 2.

Compound 5 was identified as ellagic acid based on the corresponding standards and previous literature. The parent ion [M + H]^+^ at *m/z* 303.0140 and product ions at *m/z* 245.0080 and *m/z* 201.0182 were obtained from ring opening [28]. Compounds 11, 13, and 24 were identified as isomers of ellagic acid. Compound 29 was identified as trilobatin and showed the molecular ion [M + H]^+^ at 437.1424 (C_21_H_25_O_10_^+^). The fragment ions at *m/z* 121.0635 were generated by the loss of C_6_H_10_O_5_, followed by C_7_H_6_O_4_. Compound 33 is an isomer of trilobatin and was identified as phloridzin [3]. Thus, compound 32 was identified as eriodictyol. The parent ion [M + H]^+^ was observed at *m/z* 289.0726 and the product ion *m/z* 153.0182 was generated by ring opening [3]. Compound 35 was identified as naringenin and its fragmentation pattern was similar to that of eriodictyol. Furthermore, Compound 38 was identified as β-Amyrin. The molecular ion [M + H]^+^ was obtained at 427.3914 (C_30_H_51_O^+^). The product ion *m/z* 409.3821 resulted from the loss of H_2_O, and the product ion *m/z* 219.2133 was generated by the ring opening lost by C_14_H_24_O. Compound 39 was identified as β-Amyrin acetate and compound 40 was identified as lupeol acetate.

### Differences in distribution of secondary metabolites among eight species

**Fig. 3:**
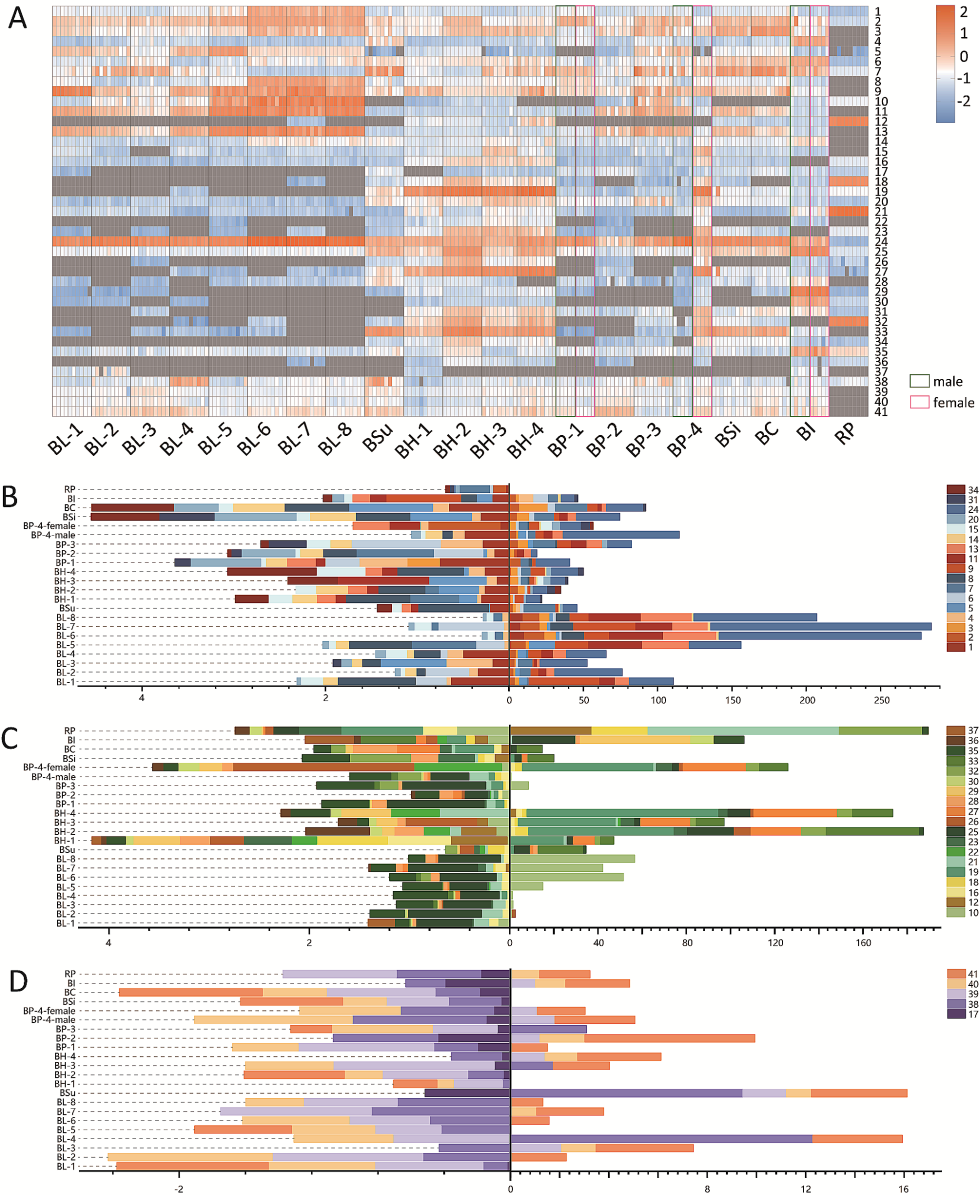
Distribution of 41 secondary metabolites in 21 batches of *Balanophoraceae* herbs. (**A**) Heatmap of 41 compounds in 21 sources of *Balanophoraceae* herbs. The figure was constructed using log10 of the average value of 10 duplicates. The level of content for each compound was indicated by colors ranging from low (blue) to high (orange), and the empty values are expressed in gray. The relative contents of (**B**) total phenolic acids and their derivatives, (**C**) total flavonoids and their derivatives, (**D**) and other compounds in 21 batches of *Balanophoraceae*

Figure 3A shows a heat map of secondary metabolites in the 209 samples, preliminarily revealing the differences or similarities between the eight *Balanophorae* species. The color of each square represents the corresponding content of each sample; orange represents an increase in concentration and blue represents a decrease in concentration. Empty values are expressed in gray. Secondary metabolites between *Balanophora* and *Rhopalocnemis* showed distinct differences. In *Balanophora*, BH contained the most types of secondary metabolites and most had higher concentrations. In the BL samples compounds 1–11 were present in considerably high concentrations. The secondary metabolites of BP had no obvious characteristics compared to the others. In addition, differences were observed among different production areas.

To further explore the differences in secondary metabolites among the eight species of *Balanophorae*, the content of each compound in the 21 batches is shown in Fig. 3B (phenolic acids and their derivatives), Fig. 3C (flavonoids and their derivatives) and Fig. 3D (others). RP showed the lowest total phenolic acid content and the highest total flavonoid content compared to *Balanophora* herbs. The flavonoid profile of BI differed from that of the other species, mainly in compounds 29, 30, and 35. The BSi samples are similar to the BC samples. For BL, BH, and BP, the total phenolic acid content in BL was higher than those in others, whereas the total flavonoids was relatively low. Among the phenolic acids, the contents of compounds 11, 13, and 24 in BL were higher than those of the other compounds. In addition, it was obvious that BL-1, 2, 3, and 4 were different from the remaining four BL batches, possibly due to the source of the samples. The secondary metabolite profile of the BP was indistinguishable from that of the BL. However, the content of compound 17 was the highest in BP, whereas the other seven batches of BL contained no compound 17, except for BL-1. In addition, the secondary metabolite profiles of BP-4 female and male plants were significantly different, and the profile of BP-4 female plants was similar to that of the BH. The secondary metabolite profile of BH showed obvious differences from those of BL and BP, which are easy to distinguish. The total flavonoid content in BH was much higher than in the other samples, mainly manifested in compounds 18, 19, 22, and 27.

### Targeted metabolomic analysis of BL, BH, and BP

**Fig. 4:**
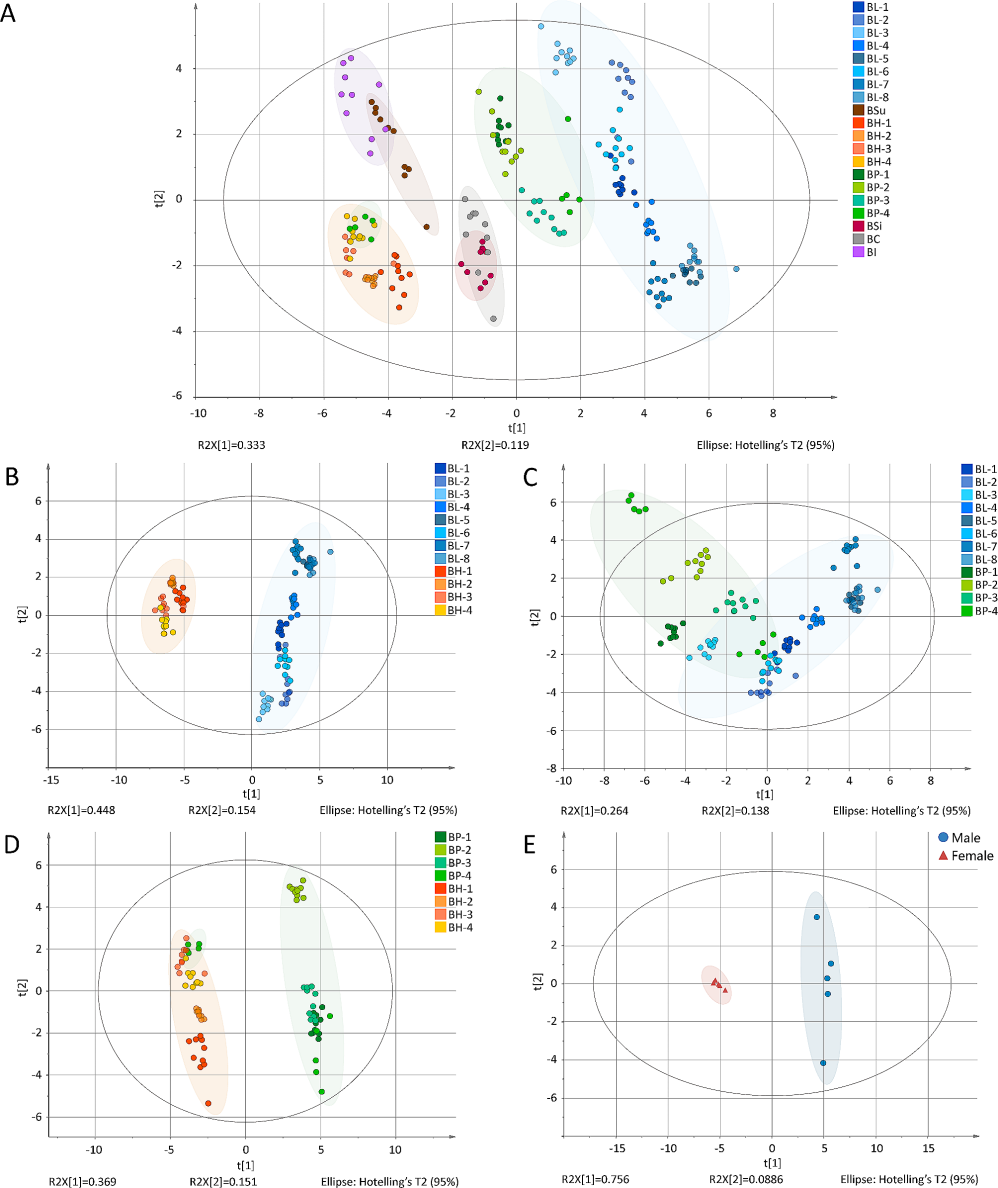
Principal component analysis (PCA) models of metabolic profiles derived from various *Balanophora* samples: (**A**) PCA scores obtained from 20 batches of *Balanophora*. (**B**) PCA scores obtained from *Balanophora laxiflora* (BL) and *Balanophora harlandii* (BH). (**C**) PCA scores obtained from BL and *Balanophora polyandra* (BP). (**D**) PCA scores obtained from BP and BH. (**E**) PCA scores obtained from male and female plants of BP-4

To explore the overall profiles among seven species of *Balanophora*, the datasets of each group were analyzed using PCA. As shown in Fig. 4A, the secondary metabolites of the samples from the seven species showed noticeable differences. However, BP-4 showed an abnormal separation, which is discussed later. Furthermore, there were differences among the different producing regions. Four batches of BH were separated into clusters of BH-1 and BH-2 and those of BH-3 and BH-4. Four batches of BL (BL-1, 2, 3, and 4) were distributed above the X-axis, and another four batches (BL-5, 6, 7, and 8) were distributed below the X-axis.

Then BL, BH, and BP were selected from several production areas for further analysis. PCA, PLS-DA, and rank sum tests were used to compare secondary metabolites among BL and BH, BL and BP, and BH and BP.

In the PCA model of BL and BH (Fig. 4B), the separation was significant (R^2^X = 0.81, Q^2^ = 0.691), indicating a difference in secondary metabolites between BL and BH. The PLS-DA model and rank-sum test were applied to further explore the differences between BL and BH. The PLS-DA model results showed R2Y and Q2 values of 0.987 and 0.986, respectively, indicating good predictability and reliability. The Q2 of the 999-time permutation tests for both PLS-DA models was negative, indicating that they were not overfitted (Figure S1A, B). As a result, 21 metabolic features with VIP > 1 and *P* < 0.05 were selected as chemical markers to distinguish BL and BH, including 8 phenolic acids and 13 flavonoids.

In the PCA model for BL and BP (Fig. 4C), the separation was significant (R^2^X = 0.876, Q^2^ = 0.647). The PLS-DA model and rank-sum test were used to explore the differences between BL and BH. The results of the PLS-DA model showed that values of R^2^Y and Q^2^ were 0.975 and 0.956, respectively, indicating that the model exhibited good prediction and reliability. Moreover, the Q^2^ of the 999-time permutation tests of the aforementioned models were negative (Figure S1C, D). Twelve metabolic features with VIP > 1 and *P* < 0.05, including nine phenolic acids, three flavonoids, and one other compound, were selected as chemical markers to distinguish between BL and BP.

In the PCA model for BH and BP (Fig. 4D), the separation was significant (R^2^X = 0.926, Q^2^ = 0.762). The results showed that although BP-4 female samples clustered with BH, BH, and BP, they still showed a certain separation. The PLS-DA model and rank-sum test were used to explore the differences between BL and BH. According to the PLS-DA model, the R2Y and Q2 were 0.970 and 0.950, respectively, indicating good prediction and reliability. Moreover, the Q^2^ of the 999-time permutation tests of the aforementioned models were negative (Figure S1E, F). As a result, 18 metabolic features with VIP > 1 and *P* < 0.05, including 3 phenolic acids and 15 flavonoids, were selected as chemical markers to distinguish between BL and BH.

The metabolic marks used to differentiate among BL and BH, BL and BP, and BH and BP are shown in Table S1. Five compounds could be used to distinguish BL, BH, and BP simultaneously, including three phenolic acids and two flavonoids.

In addition, targeted metabolomic analysis was used to explore the difference between BP-4 male and female samples (Fig. 4E). The PCA model indicated that the separation was significant (R^2^X = 0.844, Q^2^ = 0.651). The PLS-DA model showed that values of R^2^Y and Q^2^ were 0.998 and 0.992, respectively. The Q^2^ of the 999-time permutation tests of the aforementioned models were negative (Figure S1G, H). As a result, 26 metabolic features with VIP > 1 and *P* < 0.05, including 12 phenolic acids,13 flavonoids and 1 other compound, were selected as chemical markers to differentiate between male and female plants.

## Discussion

Here, comparative pharmacognosy and secondary metabolite studies were performed on dried plants of eight species of *Balanophorae*. The results revealed that eight species showed different pharmacognosy characteristics. RP and BI can be accurately identified based on their pharmacognostic characteristics. However, the origins of *Balanophorae* plants are diverse, leading to many samples not being identified by pharmacognosy. A total of 41 secondary metabolites were identified or characterized using UPLC-QTOF-MS/MS, including 17 phenolic acids and their derivatives, 19 flavonoids and their derivatives, and 5 others. There were differences in the distributions of 41 secondary metabolites among the eight species. In addition, the results of targeted metabolomic analysis showed that the secondary metabolites of the samples from the seven species had noticeable differences. Furthermore, 21, 12, and 18 metabolomic markers were screened to distinguish among BL and BH, BL and BP, and BH and BP, respectively. Among these, five important metabolic markers could simultaneously distinguish BL, BH, and BP.

Our findings indicate that the secondary metabolites of *Balanophorae* are not only affected by genes and environmental factors but also by storage factors. In a study on the distribution of secondary metabolites, the total polyphenol content of four batches of BL purchased from a medicinal market was significantly higher than that of four batches of fresh BLs. The results of the multivariate statistical analysis also showed differences. Therefore, it was inferred that changes in secondary metabolites may have been caused by the storage period. However, there are no reports on the effects of storage conditions on the chemical constituents of *Balanophorae*. A previous study reported that the total polyphenol content of apples rose to the highest level after 21 days of storage, which is consistent with the phenomenon observed in this study [29].

Targeted metabolomic analysis showed that the differential metabolites between BL and BP and BH and BP were mainly flavonoids, whereas BL and BH showed differences in total flavonoids and phenolic acids. Flavonoids possess a range of physiological and biochemical properties that allow them to participate in various interactions between plants and environmental factors. These interactions provide protection from phytophagous insects and pathogenic bacteria [30].

In addition, the secondary metabolites of BP-4 male and female plants showed significant differences, including 12 phenolic acids, 13 flavonoids, and one other compound. This is the first study to identify differences in secondary metabolites between male and female BP plants. Differences in antioxidant activity between male and female BL flowers have been reported, and this study demonstrated that crude extracts from male flowers had higher radical-scavenging activity than those from female flowers [11]. The main antioxidant substances in plants are phenolic compounds, such as phenolic acids and flavonoids. Therefore, the antioxidant activity of BP-4 in female and male plants may differ, which needs to be verified in future research.

## Conclusion

In this study, comparative pharmacognosy and secondary metabolite analyses were used to distinguish among eight species of *Balanophorae*. The pharmacognostic characteristics of the eight varieties were observed and are summarized. RP and BI can be identified based on their pharmacognostic characteristics. Then, 41 secondary metabolites were identified or characterized in the mixed extracts of the 209 samples. The distribution of secondary metabolites showed differences in the metabolite profiles among the different species. In addition, the secondary metabolites of seven species from the genus *Balanophora* showed noticeable differences in the targeted metabolomic analysis. BL, BH, and BP were successfully distinguished using metabolic markers. Five important metabolic markers that could simultaneously distinguish BL, BH, and BP were selected. These results provide valuable information for identifying diverse species of *Balanophorae* that can contribute to the quality control of related medical materials.

### Electronic supplementary material

Below is the link to the electronic supplementary material.

**Supplementary Material 1**

**Supplementary Material 2**: **Table S1** 41 compounds characterized using UPLC-QTOF-MS/MS in mixed extraction of 21 batches of materials.

**Supplementary Material 3**: **Figure S1** Partial least-squares discriminant analysis (PLS-DA) models and goodness of fit and validations (permutation tests) of the PLS-DA models. (A) PLS-DA scores and (B) scoring plots for 999-time permutation validation test of PLS-DA models that corresponded to BL and BH. (C) PLS-DA scores and (D) scoring plots for 999-time permutation validation test of PLS-DA models that corresponded to BL and BP. (E) PLS-DA scores and (F) scoring plots for 999-time permutation validation test of PLS-DA models that corresponded to BH and BP. (G) PLS-DA scores and (H) scoring plots for 999-time permutation validation test of PLS-DA models that corresponded to male and female samples of BP-4.

## Data Availability

The datasets supporting the conclusions of this study are included in this article and its additional files.

## Abbreviations

RP: *Rhopalocnemis phalloide*
BP: *Balanophora polyandra*
BL: *Balanophora laxiflora*
BH: Balanophora harlandii
LC-MS: Liquid chromatography coupled with mass spectrometry
UPLC-QTOF-MS: Ultra-high performance liquid chromatography quadrupole time-of-flight mass spectrometry
BSu: *Balanophora subcupularis*
BSi: Balanophora simaoensis
BC: *Balanophora cryptocaude*
BI: Balanophora indica
PCA: Principal component analysis
PLS-DA: Partial least-squares-discriminant analysis
EIC: Extracted ion chromatograms
