# Supplementary material for: Comparative pharmacognosy and secondary metabolite analysis of Balanophorae herbs from different sources
Source: Hereditas. 2024 Jun 21;161:19. doi: 10.1186/s41065-024-00323-1 (PMC11191205; doi:10.1186/s41065-024-00323-1)
Supplement: Supplementary file 1 — Supplementary Material 1 [file 41065_2024_323_MOESM1_ESM.pdf]

Editing Certificate

This document certifies that the manuscript listed below has been edited to ensure language and grammar accuracy and is error free in these aspects. The edit was performed by professional editors at Editage, a brand of Cactus Communications. The author's core research ideas were not altered in any way during the editing process. The quality of the edit has been guaranteed, with the assumption that our suggested changes have been accepted and the text has not been further altered without the knowledge of our editors.

MANUSCRIPT TITLE

Comparative pharmacognosy and secondary metabolite analysis of Balanophorae herbs from different sources

AUTHORS

Zhao Xueyan

ISSUED ON

April 27, 2024

JOB CODE

RNXGG_1

THE EDITAGE Promise QUALITY GUARANTEE

Prabh Grewal
Senior Vice President - Editage

editage helping you get published

Since 2002, Editage has helped over 430,000 authors publish around 1.2 million research papers in scholarly journals across over 1000 disciplines through editorial, translation, transcription, and publication support services. Editage is a brand of Cactus Communications (cactusglobal.com), a science communication and technology company.

CACTUS.

GLOBAL :
+1(833) 979-0061 | request@editage.com

CHINA :
400-120-3020或 021-6020-9400 | fabiao@editage.cn
