# Supplementary material for: Comparative pharmacognosy and secondary metabolite analysis of Balanophorae herbs from different sources
Source: Hereditas. 2024 Jun 21;161:19. doi: 10.1186/s41065-024-00323-1 (PMC11191205; doi:10.1186/s41065-024-00323-1)
Supplement: Supplementary file 2 — Supplementary Material 2: Table S1 41 compounds characterized using UPLC-QTOF-MS/MS in mixed extraction of 21 batches of materials. [file 41065_2024_323_MOESM2_ESM.doc]

**Table S1** 41 compounds characterized using UPLC-QTOF-MS/MS in mixed extraction of 21 batches of materials.

| **No.** | **RT** | **Formula** | **Name** | **[M+H]+/exact** | **[M+H]+/measure** | **PPM** | **MS/MS** |
| --- | --- | --- | --- | --- | --- | --- | --- |
| 1 ^b, d^ | 1.2 | C_14_H_10_O_8_ | Glucogallin | 333.0822 | 333.0793 | -8.71 | 171.0289 |
| 2 ^a, d^ | 1.4 | C_8_H_8_O_5_ | gallicacid | 171.0293 | 171.0262 | -18.13 | 127.0389 |
| 3 | 3.57 | C_9_H_8_O_4_ | Caffeic acid-1 | 181.0501 | 181.0489 | -6.63 | 163.0401 |
| 4 | 4.47 | C_9_H_8_O_3_ | Cinnamic acid-1 | 149.0603 | 149.0586 | -11.40 | 103.0537, 131.0509 |
| 5 ^a, b, d^ | 4.62 | C_14_H_6_O_8_ | Ellagic acid-1 | 303.0141 | 303.0140 | -0.33 | 245.008, 201.018 |
| 6 | 4.81 | C_9_H_8_O_3_ | p-Hydroxylcinnamic acid | 165.0552 | 165.0555 | 1.82 | 147.0444, 144.0444, 119.0501 |
| 7 | 5.28 | C_14_H_6_O_8_ | Methoxycinnamic acid | 179.0708 | 179.0692 | -8.94 | 149.095 |
| 8 ^d^ | 5.33 | C_10_H_10_O_4_ | Caffeic acid-2 | 181.0501 | 181.0489 | -6.63 | 163.0401 |
| 9 ^b^ | 5.36 | C_13_H_16_O_10_ | Brevifolincarboxylic acid | 293.0297 | 293.0279 | -6.14 | 247.0247, 219.0292, 191.0344 |
| 10 ^b^ | 6.52 | C_15_H_10_O_5_ | 3’, 4’-dihydroxy flavonoid-7-O-β-D-glucoside | 433.1135 | 433.1130 | -1.15 | 271.0601 |
| 11 ^a, b, c, d^ | 6.88 | C_14_H_6_O_8_ | Ellagic acid-2 | 303.0141 | 303.014 | -0.33 | 245.008, 201.018 |
| 12 ^a, c^ | 6.98 | C_21_H_22_O_11_ | Eriodictyol-glucoside-1 | 451.1240 | 451.1227 | -2.88 | 135.0194 |
| 13 ^a, b, c, d^ | 7.07 | C_14_H_6_O_8_ | Ellagic acid-3 | 303.0141 | 303.0140 | -0.33 | 245.008, 201.018 |
| 14 ^d^ | 7.11 | C_9_H_8_O_4_ | m-Hydroxylcinnamic acid | 165.0552 | 165.0555 | 1.82 | 147.0444, 144.0444, 119.0501 |
| 15 ^d^ | 7.23 | C_16_H_14_O_6_ | methyl brevifolincarboxylate | 307.0454 | 307.0452 | -0.65 | 275.0216 |
| 16 ^a, c, d^ | 7.69 | C_21_H_20_O_11_ | Luteolin-7-glucoside-1 | 449.1084 | 449.1084 | 0.00 | 287.0548 |
| 17 ^b^ | 7.88 | C_26_H_32_O_11_ | pinoresinol-4-O-glucoside | 521.2023 | 521.2000 | -4.41 | 359.1489 |
| 18 ^a, b, c^ | 8.16 | C_32_H_52_O_2_ | 5,7-dyhydroxy chromone | 179.0344 | 179.0336 | -4.47 | 153.0194 |
| 19 ^a, b, c, d^ | 8.19 | C_21_H_22_O_11_ | Eriodictyol-glucoside-2 | 451.1240 | 451.1227 | -2.88 | 135.0194 |
| 20 ^a, b, d^ | 8.23 | C7H_6_O_5_ | Methyl caffeate | 195.0657 | 195.0659 | 1.03 | 181.0489 |
| 21^c, d^ | 8.74 | C_21_H_20_O_11_ | Luteolin-7-glucoside-2 | 449.1084 | 449.1084 | 0.00 | 287.0548 |
| 22 ^a, c, d^ | 9.05 | C_21_H_24_O_11_ | 3-hydroxy-phloridzin-1 | 453.1397 | 453.1416 | 4.19 | 291.0870, 169.0490, 127.0389 |
| 23 ^a, c, d^ | 9.16 | C_21_H_20_O_11_ | Luteolin-7-glucoside-3 | 449.1084 | 449.1084 | 0.00 | 287.0548 |
| 24 ^a, b, d^ | 9.37 | C_9_H_8_O_2_ | Ellagic acid-3 | 303.0141 | 303.0140 | -0.33 | 245.008, 201.018 |
| 25 ^a, c, d^ | 9.47 | C_21_H_22_O_10_ | naringenin -glucoside-1 | 435.1291 | 435.1296 | 1.15 | 273.0739, 153.0169 |
| 26 ^a, c, d^ | 9.74 | C_22_H_24_O_11_ | Hesperetin -glucoside | 465.1378 | 465.1378 | 0.00 | 153.0194 |
| 27 ^a, c, d^ | 9.86 | C_21_H_24_O_11_ | 3-hydroxy-phloridzin-2 | 453.1397 | 453.1373 | -5.30 | 291.087, 169.0490, 127.0389 |
| 28 ^d^ | 9.99 | C_21_H_20_O_11_ | aureusidin-4-O-β-D-glucopyranoside | 449.1084 | 449.1084 | 0.00 | 287.0548 |
| 29 ^a, c, d^ | 10.38 | C_21_H_24_O_10_ | trilobatin | 437.1448 | 437.1424 | -5.49 | 121.0635 |
| 30^c, d^ | 10.64 | C_21_H_22_O_10_ | naringenin -glucoside-2 | 435.1291 | 435.1296 | 1.15 | 273.0739, 153.0169 |
| 31 ^a, b, d^ | 10.71 | C_8_H_8_O_5_ | Methyl gallate-1 | 185.0450 | 185.0438 | -6.48 | 171.0262 |
| 32 ^a, c, d^ | 11.02 | C_15_H_12_O_6_ | Eriodictyol | 289.0712 | 289.0726 | 4.84 | 153.0182 |
| 33 ^a, c, d^ | 11.47 | C_21_H_24_O_10_ | phloridzin | 437.1448 | 437.1424 | -5.49 | 121.0635 |
| 34 ^a, b, c, d^ | 12.04 | C_13_H_8_O_8_ | Methyl gallate-2 | 185.0450 | 185.0438 | -6.48 | 171.0262 |
| 35 | 12.54 | C_15_H_12_O_5_ | naringenin | 273.0763 | 273.0772 | 3.30 | 153.0194 |
| 36 ^a, c, d^ | 12.69 | C_21_H_20_O_10_ | homoeriodictyol | 303.0869 | 303.0888 | 6.27 | 153.0194 |
| 37 | 13.04 | C_9_H_6_O_4_ | 7,3',4'-Trihydroxyflavone | 271.0601 | 271.0588 | -4.80 | 135.0441, 137.0233 |
| 38 | 27.83 | C_10_H_10_O_3_ | β-Amyrin | 427.3940 | 427.3914 | -6.08 | 191.1815, 219.2113, 409.3821 |
| 39 ^d^ | 30.42 | C_30_H_50_O | β-Amyrin acetate | 469.4046 | 469.4063 | 3.62 | 191.1815, 219.2113, 409.3834 |
| 40 | 30.94 | C_32_H_52_O_2_ | Lupeol acetate-1 | 469.4046 | 469.4018 | -5.97 | 191.1815, 219.2113, 409.3834 |
| 41 | 31.19 | C_32_H_52_O_2_ | Lupeol acetate-2 | 469.4046 | 469.4018 | -5.97 | 191.1815, 219.2113, 409.3834 |

Notes: ^a^ metabolic markers screened to distinguish BL and BH; ^b^ metabolic markers metabolic markers screened to distinguish BL and BP; ^c^ metabolic markers metabolic markers screened to distinguish BH and BP; ^d^ metabolic markers metabolic markers screened to distinguish male and female samples of BP-4.
