# Supplementary material for: Comparative pharmacognosy and secondary metabolite analysis of Balanophorae herbs from different sources
Source: Hereditas. 2024 Jun 21;161:19. doi: 10.1186/s41065-024-00323-1 (PMC11191205; doi:10.1186/s41065-024-00323-1)
Supplement: Supplementary file 3 — Supplementary Material 3: Figure S1 Partial least-squares discriminant analysis (PLS-DA) models and goodness of fit and validations (permutation tests) of the PLS-DA models. (A) PLS-DA scores and (B) scoring plots for 999-time permutation validation test of PLS-DA models that corresponded to BL and BH. (C) PLS-DA scores and (D) scoring plots for 999-time permutation validation test of PLS-DA models that corresponded to BL and BP. (E) PLS-DA scores and (F) scoring plots for 999-time permutation validation test of PLS-DA models that corresponded to BH and BP. (G) PLS-DA scores and (H) scoring plots for 999-time permutation validation test of PLS-DA models that corresponded to male and female samples of BP-4. [file 41065_2024_323_MOESM3_ESM.doc]

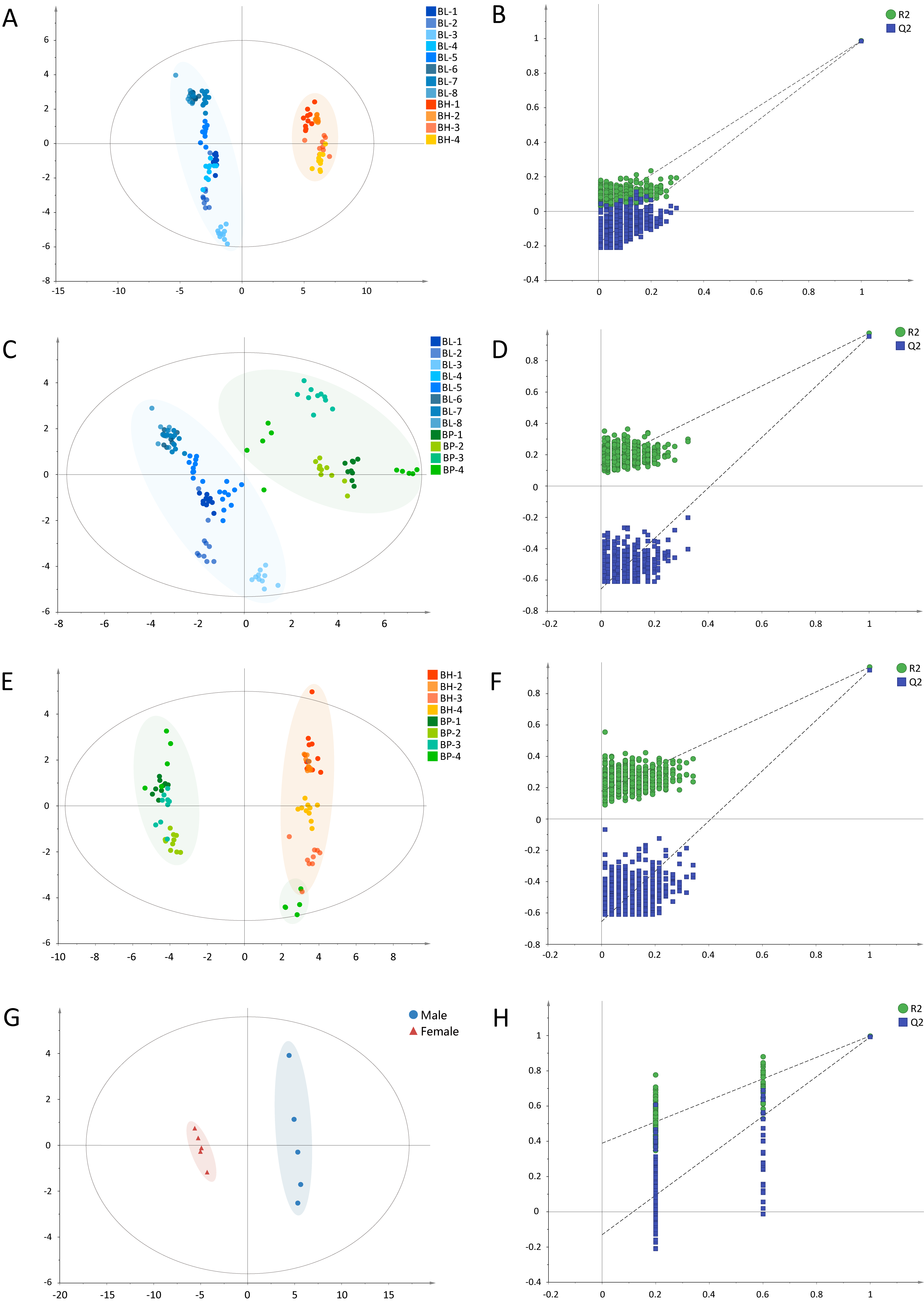


**Figure S1** Partial least-squares discriminant analysis (PLS-DA) models and goodness of fit and validations (permutation tests) of the PLS-DA models. (A) PLS-DA scores and (B) scoring plots for 999-time permutation validation test of PLS-DA models that corresponded to BL and BH. (C) PLS-DA scores and (D) scoring plots for 999-time permutation validation test of PLS-DA models that corresponded to BL and BP. (E) PLS-DA scores and (F) scoring plots for 999-time permutation validation test of PLS-DA models that corresponded to BH and BP. (G) PLS-DA scores and (H) scoring plots for 999-time permutation validation test of PLS-DA models that corresponded to male and female samples of BP-4.
